# The influence of childhood socioeconomic status on academic engagement among adolescents: the mediating role of internalization of learning motivation and learning burnout

**DOI:** 10.3389/fpsyg.2025.1641804

**Published:** 2025-11-12

**Authors:** Shuang Zhong, Guangzhe Yuan, Yanqiu Gu, Caimeng Liu

**Affiliations:** 1 Sichuan Provincial Research Center for Rural Education & Department of Educational Science, Leshan Normal University, Leshan, China; 2 The Third Middle School of Zizhong Conuty, Neijiang, China

**Keywords:** childhood SES, motivation, learning burnout, academic engagement, internalization

## Abstract

A paucity of studies has hitherto been observed in the field of research concerning the relationship between childhood socioeconomic status (SES) and academic engagement, as well as the potential mechanisms involved. The present study therefore sought to explore the effect of childhood SES on adolescents’ academic engagement, and the underlying mechanisms that may facilitate or hinder this engagement. The study was anchored in the theoretical frameworks of Self-Determination Theory and Life History Theory. A sample of 611 Chinese adolescents (Mage = 16.91, SD = 0.37, 41.2% male) were investigated in the study. The findings indicated that childhood SES had a substantial and positive influence on adolescents’ academic engagement. Furthermore, childhood SES exerts an indirect influence on adolescent academic engagement, manifesting in the internalization of learning motivation and the development of learning burnout. The hypothesis is put forward that an increase in the academic engagement of adolescents can be achieved through the promotion of learning motivation, as well as the alleviation of learning burnout.

## 1 Introduction

Academic engagement is a positive, fulfilling, and work-related state of mind that is characterized by vigor, dedication, and absorption (Sinval et al., 2021). Academic engagement has been found to be a significant predictor of students’ learning achievement and satisfaction, psychological well-being (Jin et al., 2025; Wong et al., 2024), and success in educational quality (Wang and Hofkens, 2020). For senior high school student, they tend to be more passive in learning activities and put forward more demands on students’ cognition (Juvonen, 2007), which may lead to changes in students ‘academic engagement. Previous studies have shown that students’ academic engagement is significantly correlated with their learning activities and behavior performance. Specifically, adolescent students with lower academic engagement tend to exhibit poorer academic performance, more violations and dropouts (Zhang and Lee, 2018), and higher levels of depression, anxiety and loneliness (Sasser et al., 2023). Conversely, higher levels of academic engagement are associated with higher levels of self-efficacy and subjective well-being (Pan et al., 2023; Reschly et al., 2008). Therefore, the investigation of methods to enhance students’ academic engagement and its underlying mechanisms is of significant theoretical value and practical importance for the development of a leading educational nation and the promotion of students’ personal development.

Socioeconomic status (SES) is defined as the material and non-material resources possessed by an individual and their general reflection within the social hierarchy (Bradley and Corwyn, 2002). Extensive research indicates that SES is a key predictor of individual behavior and mental health (Kong and Sun, 2021; Ridley et al., 2020). However, environmental contexts during different life stages may exert differential influences on an individual’s physical and psychological development. Belsky et al. (1991) advanced the notion that the initial familial environment exerts a considerable influence on the psychological and behavioral outcomes of individuals. Childhood SES is a core variable within the early family environment, and research indicates that it may exert a particularly prominent and lasting influence on an individual’s psychological development (Kong and Sun, 2021). A substantial body of research has indicated that individuals who have experienced poverty during their formative years are more likely to encounter elevated levels of environmental stress. This phenomenon is characterized by a tendency to exhibit behavioral problems, demonstrate a reduced adherence to social norms, and demonstrate substandard academic performance in educational settings (Belsky et al., 1991; Lurie et al., 2021). Subsequent studies have found that students with lower childhood SES also tend to display lower levels of academic engagement in school (Chang and Zhang, 2023). The present study hypothesizes that childhood SES positively associates with adolescents’ academic engagement.

As an internal drive to promote, guide and maintain students’ learning activities, learning motivation may be closely related to individual academic engagement. Self-Determination Theory (SDT) holds that individual motivation is located at a certain position on the continuum from controlled motivation to autonomous motivation, and the position of motivation is determined by the degree of motivation internalization (Deci and Ryan, 2000). The degree of motivation internalization is the result of individual self-integration through interaction with environment. Specially, social environment, such as satisfaction of basic psychological needs, can promote individual motivation internalization (Ryan and Deci, 2000). Previous studies showed that the home environment is related to children’s learning motivation (Choi and Cho, 2020; MacPhee et al., 2018). There was evidence supported that the family socioeconomic status had a significant positive correlation with students’ learning motivation (Zhou and Guo, 2009).

Despite the fact that a number of studies have examined the impact of socioeconomic status on individual academic achievement, there has been limited attention paid to the influence of childhood SES on adolescents’ academic engagement. Lindström et al. (2014) proposed that childhood SES may have a profound impact on individuals than current socioeconomic status. Life history theory (LHT) posits that individuals who experienced financial constraints during their formative years are more likely to adopt a rapid life history strategy. This entails allocating greater resources toward mating and reproduction, while reducing investment in the acquisition of knowledge and skills (Belsky, 2007; Berrigan et al., 1993). Consequently, students from lower socioeconomic backgrounds may exhibit diminished interest in learning and a weaker internalization of learning motivation. Green et al. (2012) posited that motivational factors exert a significant influence on academic engagement. In accordance with SDT, the greater the degree of internalization of individual motivation, the higher the behavioral engagement (Ryan and Deci, 2000). This conclusion is corroborated in numerous domains, including learning and sports (Fu et al., 2024; Sheldon, 2020). The present study hypothesizes that childhood SES could indirectly affect adolescent academic engagement through the internalization of learning motivation.

Learning burnout is a persistent, negative, learning-related psychological state that includes characteristics such as physical and mental exhaustion, learning detachment, and low achievement (Rho et al., 2025). According to the control-value theory of academic emotion, individual academic emotion will be influenced by family, school and other environmental factors, and then affect individual learning activities and learning outcomes (Pekrun, 2006). Based on the control-value theory of academic emotion, childhood SES may indirectly affect adolescents’ academic engagement through learning burnout. Previous studies showed that adolescent students’ family socioeconomic status significantly negative predicted learning burnout (Luo et al., 2016). However, there are few studies focused on the relationship between childhood SES and adolescent learning burnout. Therefore, the relationship between childhood SES and adolescent learning burnout is also an important issue in this study. In addition, studies have confirmed that learning burnout can negatively predicted students’ academic engagement (Liu J. et al., 2020; Jin et al., 2025; Wang et al., 2024). Therefore, this study hypothesizes that individuals with lower SES in childhood are more likely to suffer from learning burnout and thus reduce their academic engagement.

SDT offers a compelling theoretical explanation for the relationship between the internalization of learning motivation and learning burnout. According to SDT, the internalization of motivation is closely related to personal health and growth (Ryan and Deci, 2000). Research from a variety of academic disciplines has demonstrated that the internalization of motivation has a negative correlation with burnout (Hyseni et al., 2025; Labrague, 2024). Thus, the internalization of learning motivation in adolescents may serve as a predictor of learning burnout.

It has been determined that adolescents’ academic engagement is a significant predictor of both student achievement and educational quality. The extant literature has predominantly examined its antecedents in terms of teacher support, parental support, and students’ personal characteristics (Jeynes, 2024; Patall et al., 2024). However, relatively little research has been conducted on more distal factors, such as the childhood environment and broader social conditions. Moreover, there is a paucity of research on the interaction between these distal factors and individual characteristics in shaping academic engagement. The present research draws on the principles of SDT and LHT in order to investigate both distal and individual predictors of adolescents’ academic engagement. It is an irrefutable fact that human beings develop within social contexts, and their psychology and behavior are inextricably shaped by environmental forces. LHT and SDT are both concerned with the question of how context influences development. However, these two factors are not mutually exclusive and can, in fact, be considered from a complementary perspective. LHT is predicated on the premise that environmental conditions calibrate individuals’ allocation of finite resources (Peng et al., 2016), with a concomitant emphasis on developmental outcomes. Conversely, the SDT elucidates the intrinsic psychological processes through which social environments foster or impede optimal development (Deci and Ryan, 2000). Integrating these two perspectives thus provides a more comprehensive understanding of how childhood environments affect adolescents’ academic engagement and the mechanisms underlying these effects, thereby informing targeted interventions.

In summary, the four hypotheses of this study are proposed.

*Hypothesis 1*: Childhood SES positively associates with adolescents' academic engagement.

*Hypothesis 2*: Childhood SES can exert an indirect influence on adolescents' academic engagement through the internalization of learning motivation.

*Hypothesis 3*: Childhood SES indirectly influences adolescent academic engagement through learning burnout.

*Hypothesis 4*: The internalization of learning motivation negatively relates to adolescents learning burnout.

## 2 Materials and methods

### 2.1 Participants and procedure

We used cross-sectional data to analyze the relationship between childhood SES, learning motivation, learning burnout and academic engagement. Using the convenience sampling method, we recruited participants from several middle schools in China southwest. The school surveyed in this study is a regular county-level high school located in Zizhong County, Sichuan Province. A total of 656 adolescents were investigated, and a valid response was received from: *N* = 611 (Mage = 16.91, SD = 0.37). A sample of 252 subjects were identified as male, representing 41.2% of the sample. In contrast, 380 subjects were identified as female, accounting for 62.2% of the sample. With regard to geographical location, 119 subjects (19.5%) were from urban areas, and 492 (80.5%) were from rural areas. With regard to familial status, 79 subjects (12.9%) were only children, and 532 (87.1%) were not only children. With regard to the status of the subjects in relation to their parents, 380 subjects (62.2%) were identified as left-behind children, and 231 (37.8%) were not left-behind children.

The present study was approved by the Research Ethics Committee of the School of Education Science, Leshan Normal University. Consent was obtained from the school and parents before the survey began, and adolescents could withdraw at any time during the survey. Participants were required to complete self-report questionnaires by WJX which is a platform liking Mturk.

### 2.2 Measures

#### 2.2.1 Childhood SES

We used environmental harshness to access childhood SES (Griskevicius et al., 2013). The questionnaire consists of three items, each rated on a 1 (strongly disagree) to 5 (strongly agree): “My family usually had enough money for things when I was growing up,” “I grew up in a relatively wealthy neighborhood,” “I felt relatively wealthy compared to the other kids in school.” The scale has been validated in the Chinese context (Kong and Sun, 2021). In this study, the questionnaire showed great internal consistency (Cronbach’s alpha was 0.84).

#### 2.2.2 Internalization of learning motivation

The degree of internalization of learning motivation was measured using the Comprehensive Relative Autonomy Index (C-RAI, Sheldon et al., 2017). The scale is composed of six subscales: intrinsic motivation, identified motivation, positive introjected motivation, negative introjected motivation, external motivation, and amotivation. Each subscale comprises four items, thus yielding a total of 24 items. Each item is evaluated on a scale ranging from 1 (strongly disagree) to 5 (strongly agree). Examples of the items include, “I think that study is fun,” and “I would feel guilty if I did not study.” We used RAI (Relative Autonomy Index) to evaluate the internalization of learning motivation, RAI = intrinsic motivation + identified motivation + positive introjection motivation − negative introjection motivation − external motivation − amotivation. The index was computed using raw scores from the respective subscales. The high scores of RAI indicates a higher internalization of learning motivation. In this study, the scale had excellent internal consistency (Cronbach’s alpha was 0.88).

#### 2.2.3 Learning burnout

The learning burnout was measured by the Chinese version of the Maslach Burnout Inventory-Student Survey (Schaufeli et al., 2002; Wu et al., 2010). The scale under consideration comprises a total of 16 items, which are divided into three distinct scales: exhaustion (4 items), alienation (5 items), and efficacy (7 items). Each item is scored on a scale ranging from 1 (strongly disagree) to 5 (strongly agree). Examples of the statements included in the study are as follows: “I feel emotionally drained from my study” and “I do not think studying means anything to me.” The items of efficacy were reverse scored. In this study, the scale demonstrated excellent internal consistency (Cronbach’s alpha was 0.80).

#### 2.2.4 Academic engagement

Academic engagement was accessed with 17-item Utrecht Work Engagement Scale-Student (Schaufeli et al., 2002). The Chinese version of Academic Engagement Scale-Student has been validated in a sample of Chinese adolescents (Fang et al., 2008; Wan et al., 2021), which includes three dimensions: vigor (6 items), absorption (6 items), and dedication (5 items). Example of the items include, “I feel happy when I am studying intensively,” “My studies inspire me.” Each item of the scale scored on a 1 (never) to 7 (always) and higher total scores indicate more academic engagement. The scale revealed great internal consistency in this study (The Cronbach’s alpha was 0.97).

#### 2.2.5 Data analysis

In order to guarantee the validity of the responses, a series of precautionary measures were implemented prior to the distribution of the questionnaire. These precautions included the collection of anonymous data, the incorporation of lie-detection items, and the implementation of reverse scoring for a selection of items. A multicollinearity test was conducted on the model, and the results showed that the variance inflation factor (VIF) for each predictor variable ranged from 1.04 to 1.71, all below 5; the tolerance values ranged from 0.59 to 0.96, all above 0.1. This finding suggests that the model is not afflicted by multicollinearity issues.

We assessed common method bias using Harman’s single-factor test on the four questionnaires, which had a total of 61 items. The analysis yielded 11 factors with eigenvalues greater than 1, with the largest factor accounting for 27.7% of the variance. This is below the critical threshold of 40%, which is the minimum required for a meaningful analysis (Tang and Wen, 2020). A normality test was therefore conducted on the data, revealing that the variables did not conform to a normal distribution (*p* < 0.01). We then conducted preliminary descriptive analysis and Spearman correlation analysis using SPSS 22.0 (see Table 1). To ensure the accuracy of the results, it was essential to standardize all data in SPSS before conducting the mediation analysis. The hypothesized mediation model was then tested using the PROCESS 3.5 module (Hayes, 2018) in SPSS (see Table 2).

**Table 1:** Means, standard deviations, and correlations among research variables.

Research variables	M ± SD	1	2	3	4	5	6
1. Sex	0.59 ± 0.49	1					
2. Left-behind	0.62 ± 0.49	−0.029	1				
3. Childhood SES	2.58 ± 0.78	−0.077	−0.081^*^	1			
4. RAI	5.38 ± 12.32	0.109^**^	−0.065	0.092 ^*^	1		
5. Learning burnout	3.00 ± 0.51	0.041	−0.043	−0.161^***^	−0.571 ^***^	1	
6. Academic engagement	3.94 ± 1.10	0.018	−0.084^*^	0.159 ^***^	0.541 ^***^	−0.519 ^***^	1

SES, socioeconomic status; RAI represents the internalization of learning motivation *p < 0.05, **p < 0.01, ***p < 0.001.

**Table 2:** Standardized coefficients among research variables.

Outcome variable	Predictive variable	*R*	*R* ^2^	*F*	*β*	*t*
Academic engagement	Childhood SES	0.18	0.03	6.93^***^	0.15	3.68^***^
RAI	Childhood SES	0.15	0.02	4.55^**^	0.14	3.42^***^
Learning burnout	Childhood SES	0.65	0.42	109.82^***^	−0.09	−2.97^**^
	RAI				−0.62	−19.97^***^
Academic engagement	Childhood SES	0.60	0.36	67.13^***^	0.05	1.36
	RAI				0.30	7.08^***^
	Learning burnout				−0.34	−7.93^***^

For simplicity, some covariables (i.e., gender and left-behind) are not displayed. **p < 0.01, ***p < 0.001.

## 3 Results

### 3.1 Descriptive statistics and correlation analysis of variables

Table 1 showed the means, standard deviations and correlations of research variables. Results presented that childhood SES significantly correlated to the internalization of learning motivation, academic engagement, and learning burnout.

### 3.2 Mediation analysis

**Figure 1:**
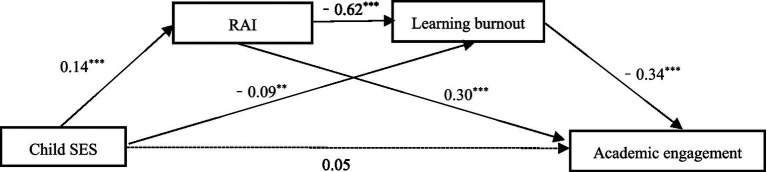
Mediation analysis path diagram. **p < 0.01, ***p < 0.001

In this study, we used model 6 in PROCESS 3.5 to access the mediation model and gender, left-behind as covariables. As demonstrated in Table 2, the results of the study indicated that the childhood SES of adolescents had a significant positive effect on their academic engagement (*β* = 0.15, *p* < 0.001). However, the direct effect was not found to be significant following the addition of the mediating variables (*β* = 0.05, *p*>0.05). Furthermore, the findings of the model demonstrated that the childhood SES of adolescents had a significant positive associated with the internalization of learning motivation (*β* = 0.14, *p* < 0.001), as well as a negative related to the level of learning burnout (*β* = −0.09, *p* < 0.01). Furthermore, the results lend support to Hypothesis 4, which posits that the degree to which learning motivation is internalized has a significantly negative effect on the occurrence of learning burnout (*β* = −0.62, *p* < 0.001) and a positive effect on academic engagement (*β* = 0.30, *p* < 0.001) (Figure 1).

In order to ascertain the significance of the mediation effect, the bootstrap method was employed for verification. The SPSS PROCESS macro was utilized to draw 5,000 repeated samples at a 95% confidence level. Table 3 showed that the total indirect effect was 0.10 [95% CI = (0.05, 0.16), *p <* 0.05]. The internalization of learning motivation mediated the association between childhood SES [indirect effect (95% CI) = 0.04 (0.01, 0.08), *p <* 0.05]; learning burnout mediated the relationship between childhood SES [indirect effect (95% CI) = 0.03(0.006, 0.06), *p <* 0.05]; The internalization of learning motivation and learning burnout fully and serially mediated the association between childhood SES [indirect effect (95% CI) = 0.03 (0.009, 0.06), *p <* 0.05].

**Table 3:** Analysis of the mediating effects in present research.

The mediating paths	Effects	Boot SE	Boot ULCI	Boot LLCI	Relative effect
Indirect effect	0.10	0.03	0.05	0.16	66.67%
Path 1	0.04	0.02	0.01	0.08	26.67%
Path 2	0.03	0.01	0.006	0.06	20%
Path 3	0.03	0.01	0.009	0.06	20%

Path 1 indicates childhood SES → internalization of learning motivation → academic engagement; Path 2 indicates childhood SES → learning burnout → academic engagement; Path 3 indicates childhood SES → internalization of learning motivation → learning burnout → academic engagement.

## 4 Discussion

The present study found a significant positive correlation between childhood SES and adolescent academic engagement, which is consistent with previous research (Chang and Zhang, 2023; Lurie et al., 2021). This finding suggests that the long-term effects of the childhood environment persist in the domain of adolescent academic engagement. This phenomenon may be attributed to the finding that a lower early family socioeconomic status tends to result in parents adopting more indifferent and neglectful parenting styles, along with increased negative responses (Bradley et al., 2001; Belsky et al., 2012). In the event of children perceiving an insecure environment within the family unit, there is a possibility that their cognitive focus will be directed toward the pursuit of immediate survival and reproduction, thus resulting in a reduction in investment in the acquisition of knowledge and skills. This phenomenon has been demonstrated to culminate in diminished academic engagement. How does childhood SES influence adolescents’ academic engagement? The present study integrated LHT and SDT in order to examine the underlying micro-level mechanisms, the results of which revealed that the effect is primarily mediated through the internalization of learning motivation and academic burnout among adolescents.

Firstly, the present study found that childhood SES influences adolescents’ academic engagement through the internalization of learning motivation, thereby supporting Hypothesis 2. While extant research based on SDT has primarily focused on the effects of parenting styles and current SES on individual learning motivation, less attention has been given to the distal factor of childhood SES. However, the findings of this study indicate that childhood SES exerts a substantial influence on adolescents’ learning motivation. According to SDT, the three basic psychological needs of humans are autonomy, competence and relatedness. The satisfaction of basic psychological needs has been demonstrated to effectively facilitate the internalization of motivation (Ryan and Deci, 2000). However, individuals who experienced poverty during childhood typically receive less material and emotional investment (Conger and Donnellan, 2007) and face more stress and social exclusion (Meng et al., 2023), resulting in lower satisfaction of their basic psychological needs and significantly impairing their motivation for future development. This motivational orientation, formed in early life, may persist into adulthood, leading to lower academic aspirations and expectations. This finding also provides a new perspective for understanding the intergenerational transmission of poverty (Yin and Li, 2021).

Secondly, the findings indicate that academic burnout plays a mediating role in the relationship between childhood SES and adolescent academic engagement. This finding provides empirical support for the Control-Value Theory of learning-related emotions (Pekrun, 2006). Individuals who experienced poverty during childhood often face increased pressure and negative emotions due to resource scarcity, leading to greater cognitive load (Shah et al., 2012). Learning is an activity that demands substantial cognitive resources. It has been demonstrated that students from lower socioeconomic backgrounds who have been subjected to chronic stress during childhood are more prone to developing negative emotions during the learning process. This necessitates an allocation of additional cognitive resources to the management of these negative emotions, consequently reducing the resources available for learning activities. Consequently, these students frequently exhibit diminished academic performance in school. Even if their family socioeconomic status improves in adulthood, the behavioral patterns and cognitive styles formed during childhood may persist and continue to exert influence, potentially negatively affecting future work engagement and even family involvement. The findings of this study provide novel insights into the academic achievements of students from economically disadvantaged backgrounds.

Finally, childhood SES has the capacity to exert influence on adolescents’ academic engagement, operating via a sequential mediation process involving learning motivation and academic burnout. This finding is consistent with the conclusions of earlier research (Hyseni et al., 2025; Labrague, 2024; Sheldon et al., 2017). Whilst preceding studies have investigated the multifaceted impacts of childhood SES on individual physical and psychological development, research on the relationships and mechanisms between these outcomes has been limited. The findings of this study demonstrate the presence of a distinct mediating pathway: childhood SES → internal motivation → emotional experience → academic engagement. Moreover, the mediation analysis suggests that childhood SES does not directly impact academic engagement; rather, it functions through individual learning motivation and negative emotions experienced during the learning process. This suggests that even individuals who have experienced adverse childhood environments have the capacity to enhance their academic engagement by improving support systems, cultivating interest in learning (Jeynes, 2024; Patall et al., 2024), and other means that promote learning motivation and alleviate academic burnout.

### 4.1 Limitations and implications

The present study is subject to several limitations. Firstly, the utilization of cross-sectional data to investigate the association between childhood SES and adolescents’ academic engagement precludes the ability to make causal inferences between variables, thereby necessitating caution when generalizing these findings. Secondly, the assessment of childhood SES primarily relied on childhood environment indicators, without incorporating factors such as parental education, occupation, and annual household income, which may affect the external validity of the results. Finally, extant research indicates that current socioeconomic status influences students’ academic engagement (Marks, 2000; Poon, 2020). However, this variable was not controlled for in the present study, potentially impacting the accuracy of the findings.

Moreover, the findings of this study offer insights that could inform strategies to enhance adolescents’ academic engagement. Firstly, it is imperative to enhance policy support for students from economically disadvantaged families. Evidence has demonstrated that the provision of tuition waivers and material assistance to children under 12 can help to secure their basic learning needs, reduce life stressors and safeguard their education. Secondly, although childhood SES is unchangeable for middle school and university students, academic engagement can still be enhanced by increasing parental and teachers’ support, as well as through school education (Liu H. et al., 2020), in order to fulfil students’ basic psychological needs and foster learning motivation. Finally, empirical evidence suggests that meditation and mindfulness can effectively alleviate negative emotions, enhance concentration, and promote well-being (Sun and Li, 2022). It is an established fact that children from low-income families are more prone to experience negative emotions as a result of elevated stress levels. Consequently, educational institutions may consider integrating mental health services, such as meditation and mindfulness training, into their curriculum to enhance students’ emotional regulation skills and alleviate symptoms of academic burnout.

## 5 Conclusion

The current study integrates the LHT and SDT to elucidate the psychological pathways that underpin the influence of childhood SES on adolescents’ academic engagement. The findings of this study demonstrate how early environmental conditions, conceptualized through the lens of LHT, fundamentally shape learning engagement via the mediating roles of motivational internalization and academic burnout, both core constructs within SDT. The integration of these theoretical concepts has resulted in the development of a novel framework that effectively elucidates the transformation of distal socioeconomic factors into proximal educational outcomes through intrinsic psychological processes.

## Data Availability

The raw data supporting the conclusions of this article will be made available by the authors, without undue reservation.

## References

[ref1] Belsky J. (2007). “Childhood experiences and reproductive strategies” in The Oxford handbook of evolutionary psychology. eds. Dunbar R. Barrett L. (England: Oxford University Press), 237–254.

[ref2] Belsky J. Schlomer G. L. Ellis B. J. (2012). Beyond cumulative risk: distinguishing harshness and unpredictability as determinants of parenting and early life history strategy. Dev. Psychol. 48, 662–673. doi: 10.1037/a0024454, PMID: 21744948

[ref3] Belsky J. Steinberg L. Draper P. (1991). Childhood experience, interpersonal development and reproductive strategy: an evolutionary theory of socialization. Child Dev. 62, 647–670. doi: 10.2307/1131166, PMID: 1935336

[ref4] Berrigan D. Charnov E. L. Purvis A. Harvey P. H. (1993). Phylogenetic contrasts and the evolution of mammalian life histories. Evol. Ecol. 7, 270–278. doi: 10.1007/BF01237647

[ref5] Bradley R. H. Corwyn R. F. (2002). Socioeconomic status and child development. Annu. Rev. Psychol. 53, 371–399. doi: 10.1177/1063426611421007 11752490

[ref6] Bradley R. H. Corwyn R. F. McAdoo H. P. Coll C. G. (2001). The home environments of children in the United States part I: variations by age, ethnicity, and poverty status. Child Dev. 72, 1844–1867. doi: 10.1111/1467-8624.t01-1-00382 11768149

[ref7] Chang B. Zhang J. (2023). The effect of childhood SES on academic engagement among secondary vocational student: the role of basic psychological needs satisfaction, and growth mindset. J. Mudanjiang Normal Coll. 234, 83–93. doi: 10.13815/j.cnki.jmtc(pss).2023.02.007

[ref8] Choi N. Cho H. J. (2020). Temperament and home environment characteristics as predictors of young children’s learning motivation. Early Child. Educ. J. 48, 607–620. doi: 10.1007/s10643-020-01019-7

[ref9] Conger R. D. Donnellan M. B. (2007). An interactionist perspective on the socioeconomic context of human development. Annu. Rev. Psychol. 58, 175–199. doi: 10.1146/annurev.psych.58.110405.085551, PMID: 16903807

[ref10] Deci E. L. Ryan R. M. (2000). The" what" and" why" of goal pursuits: human needs and the self-determination of behavior. Psychol. Inq. 11, 227–268. doi: 10.1207/S15327965PLI1104_01

[ref11] Fang L. Shi K. Zhang F. (2008). Reliability and validity of the Chinese version of the learning commitment scale. Chin. J. Clin. Psychol. 16, 618–620. doi: 10.16128/j.cnki.1005-3611.2008.06.023

[ref12] Fu P. Gao C. Chen X. Zhang Z. Chen J. Yang D. (2024). Proactive personality and its impact on online academic engagement through positive emotions and learning motivation. Sci. Rep. 14, 1–11. doi: 10.1038/s41598-024-79776-3 39548208 PMC11568340

[ref13] Green J. Liem G. A. D. Martin A. J. Colmar S. Marsh H. W. McInerney D. (2012). Academic motivation, self-concept, engagement, and performance in high school: key processes from a longitudinal perspective. J. Adolesc. 35, 1111–1122. doi: 10.1016/j.adolescence.2012.02.016, PMID: 22460236

[ref15] Griskevicius V. Ackerman J. M. Cantú S. M. Delton A. W. Robertson T. E. Simpson J. A. (2013). When the economy falters, do people spend or save? Responses to resource scarcity depend on childhood environments. Psychol. Sci. 24, 197–205. doi: 10.1177/0956797612451471 23302295

[ref16] Hayes A. F. (2018). Introduction to mediation, moderation, and conditional process analysis, second edition. New York: Guilford Press.

[ref17] Hyseni D. Z. Jahiu G. Geci D. (2025). The interplay of individual and organizational factors with early childhood teachers’ level of work motivation, job satisfaction, and burnout. Int. J. Educ. Reform 34, 106–121. doi: 10.1177/10567879221114891

[ref18] Jeynes W. H. (2024). A meta-analysis: the association between relational parental involvement and student and parent outcome variables. Educ. Urban Soc. 56, 564–600. doi: 10.1177/00131245231179674

[ref19] Jin G. Wang Q. Lei J. Chen Y. Liu S. (2025). The relationship between effort-reward imbalance and academic engagement: the chain-mediating role of academic self-concept and academic burnout. Psychol. Sch. 62, 899–907. doi: 10.1002/pits.23363

[ref20] Juvonen J. (2007). Reforming middle schools: focus on continuity, social connectedness, and engagement. Educ. Psychol. 42, 197–208. doi: 10.1080/00461520701621046

[ref21] Kong Y. Sun S. (2021). Childhood SES, life history strategy and consumption: China traditional values of "unity and harmony" as moderator. Psychol. Sci. 44, 126–133. doi: 10.16719/j.cnki.1671-6981.20210118

[ref22] Labrague L. J. (2024). The impact of job burnout on nurses' caring behaviors: exploring the mediating role of work engagement and job motivation. Int. Nurs. Rev. 71, 653–660. doi: 10.1111/inr.12899, PMID: 37908133

[ref23] Lindström M. Fridh M. Rosvall M. (2014). Economic stress in childhood and adulthood, and poor psychological health: three life course hypotheses. Psychiatry Res. 215, 386–393. doi: 10.1016/j.psychres.2013.11.018, PMID: 24332463

[ref24] Liu J. Peng P. Luo L. (2020). The relation between family socioeconomic status and academic achievement in China: a meta-analysis. Educ. Psychol. Rev. 32, 49–76. doi: 10.1007/s10648-019-09494-0

[ref25] Liu H. Yao M. Li J. (2020). Chinese adolescents' achievement goal profiles and their relation to academic burnout, academic engagement, and test anxiety. Learn. Individ. Differ. 83:101945. doi: 10.1016/j.lindif.2020.101945

[ref26] Luo Y. Wang Z. Zhang H. Chen A. (2016). The influence of family socio-economic status on learning burnout in adolescents: mediating and moderating effects. J. Child Fam. Stud. 25, 2111–2119. doi: 10.1007/s10826-016-0400-2

[ref27] Lurie L. A. Hagen M. P. McLaughlin K. A. Sheridan M. A. Meltzoff A. N. Rosen M. L. (2021). Mechanisms linking socioeconomic status and academic achievement in early childhood: cognitive stimulation and language. Cogn. Dev. 58:101045. doi: 10.1016/j.cogdev.2021.101045, PMID: 33986564 PMC8112571

[ref1002] MacPhee D. Prendergast S. Albrecht E. Walker A. K. Miller-Heyl J. (2018). The child-rearing environment and children’s mastery motivation as contributors to school readiness. J. Appl. Dev. Psychol. 56, 1–12. doi: 10.1016/j.appdev.2018.01.003, PMID: 33986564

[ref28] Marks H. M. (2000). Student engagement in instructional activity: patterns in the elementary, middle, and high school years. Am. Educ. Res. J. 37, 153–184. doi: 10.2307/1163475

[ref29] Meng K. Li F. Wang L. Chen M. (2023). Childhood SES and mental health of rural adult residents: the roles of hope and subjective well-being. J. Psychol. Sci. 46, 1148–1155. doi: 10.16719/j.cnki.1671-6981.20230515

[ref30] Pan Z. Wang Y. Derakhshan A. (2023). Unpacking Chinese EFL students’ academic engagement and psychological well-being: the roles of language teachers’ affective scaffolding. J. Psycholinguist. Res. 52, 1799–1819. doi: 10.1007/s10936-023-09974-z, PMID: 37249799

[ref31] Patall E. A. Yates N. Lee J. Chen M. Bhat B. H. Lee K. . (2024). A meta-analysis of teachers’ provision of structure in the classroom and students’ academic competence beliefs, engagement, and achievement. Educ. Psychol. 59, 42–70. doi: 10.1080/00461520.2023.2274104

[ref32] Pekrun R. (2006). The control-value theory of achievement emotions: assumptions, corollaries, and implications for educational research and practice. Educ. Psychol. Rev. 18, 315–341. doi: 10.1007/s10648-006-9029-9

[ref33] Peng Y. Wang X. Wu S. Jin S. Sun R. (2016). A brief introduction of life history theory and its combination with social psychology: moral behaviors as an example. Adv. Psychol. Sci. 24, 464–474. doi: 10.3724/SP.J.1042.2016.00464

[ref34] Poon K. (2020). The impact of socioeconomic status on parental factors in promoting academic achievement in Chinese children. Int. J. Educ. Dev. 75:102175. doi: 10.1016/j.ijedudev.2020.102175

[ref35] Reschly A. L. Huebner E. S. Appleton J. J. Antaramian S. (2008). Engagement as flourishing: the contribution of positive emotions and coping to adolescents' engagement at school and with learning. Psychol. Sch. 45, 419–431. doi: 10.1002/pits.20306

[ref36] Rho J. Nam G. Shin Y. Byeon Y. Lee J. Kim Y. . (2025). Exploring the interplay of personality traits, academic burnout, and academic engagement in dental students. BMC Med. Educ. 25:593. doi: 10.1186/s12909-024-06479-8, PMID: 40269890 PMC12016419

[ref37] Ridley M. Rao G. Schilbach F. Patel V. (2020). Poverty, depression, and anxiety: causal evidence and mechanisms. Science 370:eaay0214. doi: 10.1126/science.aay0214, PMID: 33303583

[ref38] Ryan R. M. Deci E. L. (2000). Self-determination theory and the facilitation of intrinsic motivation, social development, and well-being. Am. Psychol. 55, 68–78. doi: 10.1037//0003-066x.55.1.68, PMID: 11392867

[ref39] Sasser J. Li C. Doane L. D. Krasnow A. Murugan V. Magee D. M. . (2023). Associations between COVID-19 sleep patterns, depressive symptoms, loneliness, and academic engagement: a latent profile analysis. J. Am. Coll. Heal. 73, 1168–1172. doi: 10.1080/07448481.2023.2239361, PMID: 37535853

[ref40] Schaufeli W. B. Martinez I. M. Pinto A. M. Salanova M. Bakker A. B. (2002). Burnout and engagement in university students: a cross-national study. J. Cross-Cult. Psychol. 33, 464–481. doi: 10.1177/0022022102033005003

[ref41] Shah A. K. Mullainathan S. Shafir E. (2012). Some consequences of having too little. Sci. 338, 682–685. doi: 10.1126/science.1222426, PMID: 23118192

[ref42] Sheldon K. M. Osin E. N. Gordeeva T. O. Suchkov D. D. Sychev O. A. (2017). Evaluating the dimensionality of self-determination theory's relative autonomy continuum. Personal. Soc. Psychol. Bull. 43, 1215–1238. doi: 10.1177/0146167217711915, PMID: 28903685

[ref1001] Sheldon K. M. (2020). Going the distance on the Pacific Crest Trail: the vital role of identified motivation. Motiv. Sci. 6, 177–181. doi: 10.1037/mot0000147

[ref43] Sinval J. Casanova J. R. Marôco J. Leandro S. (2021). University student engagement inventory (USEI): psychometric properties. Curr. Psychol. 40, 1608–1620. doi: 10.1007/s12144-018-0082-6

[ref44] Sun S. Li X. (2022). The safety of meditation. Adv. Psychol. Sci. 30, 2570–2585. doi: 10.3724/SP.J.1042.2022.02570

[ref45] Tang D. Wen Z. (2020). Statistical approaches for testing common method bias: problems and suggestions. J. Psychol. Sci. 43, 215–223. doi: 10.16719/j.cnki.1671-6981.20200130

[ref46] Wan J. Zhang Y. Zhao Y. (2021). The relationship between sense of security and learning engagement of rural left-behind junior high students: the chain mediating role of academic self-efficacy and internet addiction. Chin. J. Spec. Educ. 11, 83–89.

[ref47] Wang M. Hofkens T. L. (2020). Beyond classroom learnings: a school-wide and multi-contextual perspective on student engagement in school. Adolesc. Res. Rev. 5, 419–433. doi: 10.1007/s40894-019-00115-z 33313381 PMC7732163

[ref48] Wang X. Zhang M. Wang J. (2024). The effect of university students’ academic self-efficacy on academic burnout: the chain mediating role of intrinsic motivation and academic engagement. J. Psychoeduc. Assess. 42, 798–812. doi: 10.1177/07342829241252863

[ref49] Wong Z. Liem G. A. D. Chan M. Datu J. A. D. (2024). Student engagement and its association with academic achievement and subjective well-being: a systematic review and meta-analysis. J. Educ. Psychol. 116, 48–75. doi: 10.1037/edu0000833

[ref50] Wu Y. Dai X. Wen Z. (2010). The development of adolescent student burnout inventory. Chin. J. Clin. Psychol. 18, 152–154. doi: 10.16128/j.cnki.1005-3611.2010.02.018

[ref51] Yin X. Li Z. (2021). Research on poverty and its psychological transmission between generations. J. South China Agric. Univ. (Soc. Sci. Ed.) 20, 1–13.

[ref52] Zhang Z. Lee C. (2018). Students' behavioral and emotional participation in learning activities in the mathematics classroom: a multilevel confirmatory factor analysis. J. Exp. Educ. 86, 610–632. doi: 10.1080/00220973.2017.1335684

[ref53] Zhou S. Guo Y. (2009). Analysis on correlation between middle school students’ study motivation and family economic condition. Educ. Res. Mon. 11, 23–26+35. doi: 10.16477/j.cnki.issn1674-2311.2009.11.016

